# Effects of computerized clinical decision support systems on practitioner performance and patient outcomes: Methods of a decision-maker-researcher partnership systematic review

**DOI:** 10.1186/1748-5908-5-12

**Published:** 2010-02-05

**Authors:** R Brian Haynes, Nancy L Wilczynski

**Affiliations:** 1 Health Information Research Unit, Department of Clinical Epidemiology and Biostatistics, McMaster University, Health Sciences Centre, 1280 Main Street West, Hamilton, Ontario, Canada

## Abstract

**Background:**

Computerized clinical decision support systems are information technology-based systems designed to improve clinical decision-making. As with any healthcare intervention with claims to improve process of care or patient outcomes, decision support systems should be rigorously evaluated before widespread dissemination into clinical practice. Engaging healthcare providers and managers in the review process may facilitate knowledge translation and uptake. The objective of this research was to form a partnership of healthcare providers, managers, and researchers to review randomized controlled trials assessing the effects of computerized decision support for six clinical application areas: primary preventive care, therapeutic drug monitoring and dosing, drug prescribing, chronic disease management, diagnostic test ordering and interpretation, and acute care management; and to identify study characteristics that predict benefit.

**Methods:**

The review was undertaken by the Health Information Research Unit, McMaster University, in partnership with Hamilton Health Sciences, the Hamilton, Niagara, Haldimand, and Brant Local Health Integration Network, and pertinent healthcare service teams. Following agreement on information needs and interests with decision-makers, our earlier systematic review was updated by searching Medline, EMBASE, EBM Review databases, and Inspec, and reviewing reference lists through 6 January 2010. Data extraction items were expanded according to input from decision-makers. Authors of primary studies were contacted to confirm data and to provide additional information. Eligible trials were organized according to clinical area of application. We included randomized controlled trials that evaluated the effect on practitioner performance or patient outcomes of patient care provided with a computerized clinical decision support system compared with patient care without such a system.

**Results:**

Data will be summarized using descriptive summary measures, including proportions for categorical variables and means for continuous variables. Univariable and multivariable logistic regression models will be used to investigate associations between outcomes of interest and study specific covariates. When reporting results from individual studies, we will cite the measures of association and p-values reported in the studies. If appropriate for groups of studies with similar features, we will conduct meta-analyses.

**Conclusion:**

A decision-maker-researcher partnership provides a model for systematic reviews that may foster knowledge translation and uptake.

## Background

Computerized clinical decision support systems (CCDSSs) are information technology-based systems designed to improve clinical decision-making. Characteristics of individual patients are matched to a computerized knowledge base, and software algorithms generate patient-specific information in the form of assessments or recommendations. As with any healthcare intervention with claims to improve healthcare, CCDSSs should be rigorously evaluated before widespread dissemination into clinical practice. Further, for CCDSSs that have been properly evaluated for clinical practice effects, a process of 'knowledge translation' (KT) is needed to ensure appropriate implementation, including both adoption if the findings are positive and foregoing adoption if the trials are negative or indeterminate.

The Health Information Research Unit (HIRU) at McMaster University has previously completed highly cited systematic reviews of trials of all types of CCDSSs [1-3]. The most recent of these [1] included 87 randomized controlled trials (RCTs) and 13 non-randomized trials of CCDSSs, published up to September 2004. This comprehensive review found some evidence for improvement of the processes of clinical care across several types of interventions. The evidence summarized in the review was less encouraging in documenting benefits for patients: only 52 of the 100 trials included a measure of clinical outcomes and only seven (13%) of these reported a statistically significant patient benefit. Further, most of the effects measured were for 'intermediate' clinical variables, such as blood pressure and cholesterol levels, rather than more patient-important outcomes. However, most of the studies were underpowered to detect a clinically important effect. The review assessed study research methods and, fortunately, found study quality improved over time.

We chose an opportunity for 'KT synthesis' funding from the Canadian Institutes of Health Research (CIHR) to update the review, partnering with our local hospital administration and clinical staff and our regional health authority. We are in the process of updating this review and, in view of the large number of trials and clinical applications, split it into six reviews: primary preventive care, therapeutic drug monitoring and dosing, drug prescribing, chronic disease management, diagnostic test ordering and interpretation, and acute care management. The timing of this update and separation into types of application were auspicious considering the maturation of the field of computerized decision support, the increasing availability and sophistication of information technology in clinical settings, the increasing pace of publication of new studies on the evaluation of CCDSSs, and the plans for major investments in information technology (IT) and quality assurance (QA) in our local health region and elsewhere. In this paper, we describe the methods undertaken to form a decision-maker-research partnership and update the systematic review.

## Methods

**Figure 1:**
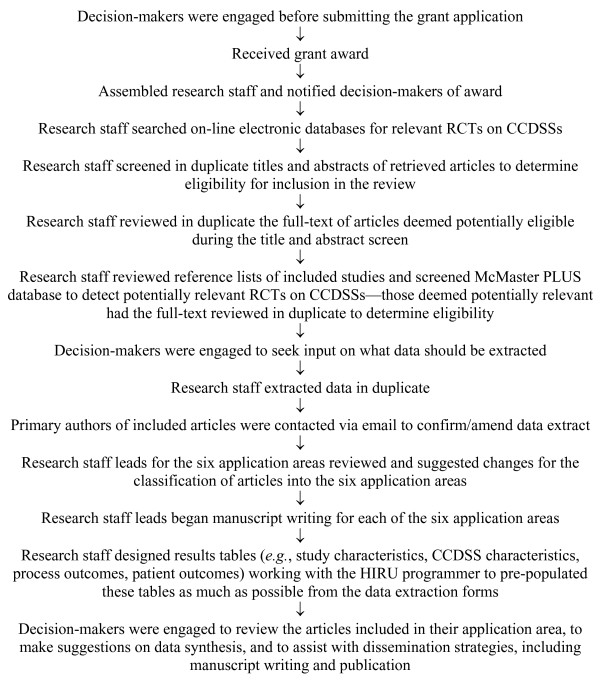
**Flow diagram of steps involved in conducting this review**.

Steps involved in conducting this update are shown in Figure 1.

### Research questions

Research questions were agreed upon by the partnership (details below). For each of the six component reviews, we will determine whether the accumulated trials for that category show CCDSS benefits for practitioner performance or patient outcomes. Additionally, conditional on a positive result for this first question for each component review, we will determine which features of the successful CCDSSs lend themselves to local implementation. Thus, the primary questions for this review are: Do CCDSSs improve practitioner performance or patient outcomes for primary preventive care, therapeutic drug monitoring and dosing, drug prescribing, chronic disease management, diagnostic test ordering and interpretation, and acute care management? If so, what are the features of successful systems that lend themselves to local implementation?

CCDSSs were defined as information systems designed to improve clinical decision-making. A standard CCDSS can be broken down into the following components. First, practitioners, healthcare staff, or patients can manually enter patient characteristics into the computer system, or alternatively, electronic medical records can be queried for retrieval of patient characteristics. The characteristics of individual patients are then matched to a computerized knowledge base (expert physician opinion or clinical practice guidelines usually form the knowledge base for a CCDSS). Next, the software algorithms of the CCDSS use the patient information and knowledge base to generate patient-specific information in the form of assessments (management options or probabilities) and/or recommendations. The computer-generated assessments or recommendations are then delivered to the healthcare provider through various means, including a computer screen, the electronic medical record, by pager, or printouts placed in a patient's paper chart. The healthcare provider then chooses whether or not to employ the computer-generated recommendations.

### Partnering with decision-makers

For this synthesis project, HIRU partnered with the senior administration of Hamilton Health Sciences (HHS, one of Canada's largest hospitals), our regional health authority (the Hamilton, Niagara, Haldimand, and Brant Local Health Integration Network (LHIN)), and clinical service chiefs at local hospitals. The partnership recruited leading local and regional decision-makers to inform us of the pertinent information to extract from studies from their perspectives as service providers and managers. Our partnership model was designed to facilitate KT, that is, to engage the decision-makers in the review process and feed the findings of the review into decisions concerning IT applications and purchases for our health region and its large hospitals.

The partnership model has two main groups. The first group is the decision-makers from the hospital and region and the second is the research staff at HIRU at McMaster University. Each group has a specific role. The role of the decision-makers is to guide the review process. Two types of decision-makers are being engaged. The first type provides overall direction. The names and positions of these decision-makers are shown in Table 1. The second type of decision-maker provides specific direction for each of the six clinical application areas of the systematic review. These decision-makers are shown in Table 2. Each of these clinical service decision-makers (shown in Table 2) is partnered with a research staff lead for each of the six component reviews. The role of the research staff is to do the work 'in the trenches,' that is, undertake a comprehensive literature search, extract the data, synthesize the data, plan dissemination, and engage in the partnership. This group is comprised of physicians, pharmacists, research staff, graduate students, and undergraduate students. The partners will continue to work together throughout the review process.

**Table 1:** Name and position of decision-makers providing overall direction

Decision-maker	Position
Murray Glendining	Executive Vice President Corporate Affairs Hamilton Health Sciences; Chief Information Officer for LHIN4

Akbar Panju	Co-chair LHIN4 implementation committee for chronic disease management and prevention

Rob Lloyd	Director, Medical Informatics Hamilton Health Sciences

Chris Probst	Director, Clinical Informatics Hamilton Health Sciences

Teresa Smith	Director, Quality Assurance, Quality Improvement Hamilton Health Sciences

Wendy Gerrie	Director, Decision Support Services Hamilton Health Sciences

**Table 2:** Name and position of decisions makers for each of the six clinical application areas

Clinical Application Area	Decision-maker	Position
Primary preventive care	Rolf Sebaldt	Director, Clinical Data Systems and Management Group McMaster University

Therapeutic drug monitoring and dosing	Stuart Connolly	Director, Division of Cardiology Hamilton Health Sciences

Drug prescribing	Anne Holbrook Marita Tonkin	Director, Division of Clinical Pharmacology and Therapeutics McMaster University Director, Chief of Pharmacy Practice Hamilton Health Sciences

Chronic disease management	Hertzel Gerstein Rolf Sebaldt	Director, Diabetes Care and Research Program Hamilton Health Sciences Director, Clinical Data Systems and Management Group McMaster University

Diagnostic test ordering and interpretation	David Koff John You	Chief, Department of Diagnostic Imaging Hamilton Health Sciences Department of Medicine McMaster University

Acute care management	Rob Lloyd	Medical Director, Pediatric Intensive Care Unit Hamilton Health Sciences

Both types of decision-makers were engaged early in the review process. Their support was secured before submitting the grant application. Each decision-maker partner was required by the funding agency, CIHR, to sign an acknowledgement page on the grant application and provide a letter of support and curriculum vitae. Research staff in HIRU met with each of the clinical service decision makers independently, providing them with copies of the data extraction form used in the previous review and sample articles in their content areas, to determine what data should be extracted from each of the included studies. Specifically, we asked them to tell us what information from such investigations they would need when deciding about implementation of computerized decision support.

Engaging the decision-makers at the data extraction stage was enlightening, and let us know that decision-makers are interested in, among other things:

1. Implementation challenges, for example, how was the system put into place? Was it too cumbersome? Was it too slow? Was it part of an electronic medical record or computerized physician order entry system? How did it fit into existing workflow?

2. Training details, for example, how much training on the use of the CCDSS was done, by whom, and how?

3. The evidence base, for example, if and how the evidence base for decision support was maintained?

4. Customization, for example, was the decision support system customizable?

All of this led to richer data extraction to be undertaken for those CCDSSs that show benefit.

We continued to engage the decision-makers throughout the review process by meeting with them once again before data analysis to discuss how best to summarize the data and to determine how to separate the content into the six component reviews. Prior to manuscript submission, decision-makers will be engaged in the dissemination phase, engaging in manuscript writing and authorship of their component reviews.

### Studies eligible for review

As of 13 January 2010 we started with 86 CCDSS RCTs identified in our previously published systematic review [1] (one of the 87 RCTs from the previous review was excluded because the CCDSS did not provide patient-specific information), and exhaustive searches that were originally completed in September 2004 were extended and updated to 6 January 2010. Consideration was given only to RCTs (including cluster RCTs), given that participants in CCDSS trials generally cannot be blinded to the interventions and RCTs at least assure protection from allocation bias. For this update, we included RCTs in any language that compared patient care with a CCDSS to routine care without a CCDSS and evaluated clinical performance (*i.e*., a measure of process of care) or a patient outcome. Additionally, to be included in the review, the CCDSS had to provide patient-specific advice that was reviewed by a healthcare practitioner before any clinical action. CCDSSs for all purposes were included in the review. Studies were excluded if the system was used solely by students, only provided summaries of patient information, provided feedback on groups of patients without individual assessment, only provided computer-aided instruction, or was used for image analysis.

The five questions answered to determine if a study was eligible for inclusion in the review were:

1. Is this study focused on evaluating a CCDSS?

2. Is the study a randomized, parallel controlled trial (not randomized time-series) where patient care with a CCDSS is compared to patient care without a CCDSS?

3. Is the CCDSS used by a healthcare professional-physicians, nurses, dentists, *et al*.-in a clinical practice or post-graduate training (not studies involving only students and not studies directly influencing patient decision making)?

4. Does the CCDSS provide patient-specific information in the form of assessments (management options or probabilities) and/or recommendations to the clinicians?

5. Is clinical performance (a measure of process of care) and/or patient outcomes (on non-simulated patients) (including any aspect of patient well-being) described?

A response of 'yes' was required for all five questions for the article to be considered for inclusion in the review.

### Finding Relevant Studies

We have previously described our methods of finding relevant studies until 2004 [1]. An experienced librarian developed the content terms for the search filters used to identify clinical studies of CCDSSs. We pilot tested the search strategies and modified them to ensure that they identified known eligible articles. The search strategies used are shown in the Appendix. For this update, we began by examining citations retrieved from Medline, EMBASE, Ovid's Evidence-Based Medicine Reviews database (includes Cochrane Database of Systematic Reviews, ACP Journal Club, Database of Abstracts of Reviews of Effects (DARE), Cochrane Central Register of Controlled Trials (CENTRAL/CCTR), Cochrane Methodology Register (CMR), Health Technology Assessments (HTA), and NHS Economic Evaluation Database (NHSEED)), and Inspec bibliographic database from 1 January 2004 to 6 January 2010. The search update was initially conducted from January 1, 2004 to December 8, 2008, and subsequently to January 6, 2010. The numbers of citations retrieved from each database are shown in the Appendix. All citations were uploaded into an in-house literature evaluation software system.

**Figure 2:**
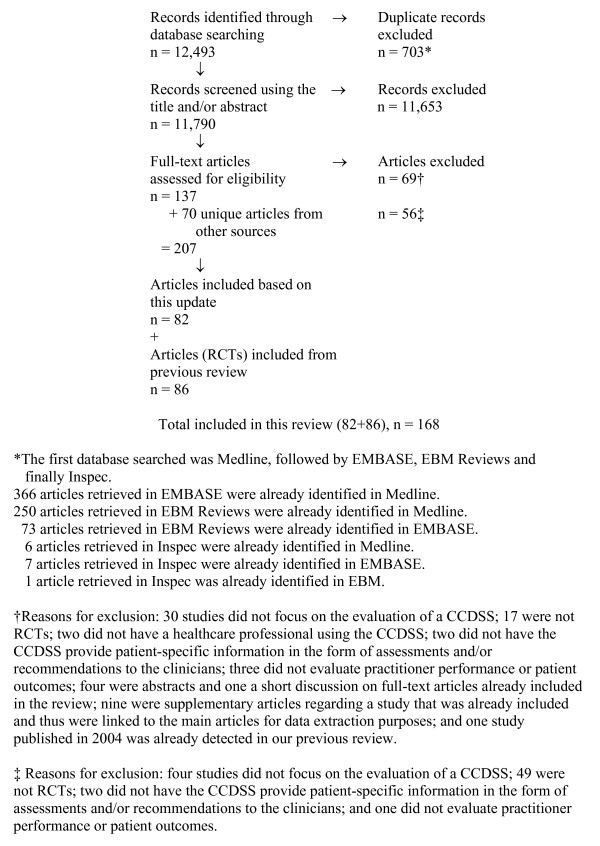
**Flow diagram of included and excluded studies for the update January 1, 2004 to December 8, 2008 as of January 13, 2010 (Number for the further update to January 6, 2010 will appear in the individual clinical application results papers)**.

Pairs of reviewers independently evaluated the eligibility of all studies identified in our search. Disagreements were resolved by a third reviewer. Full-text articles were retrieved for articles where there was a disagreement. Supplementary methods of finding studies included a review of included article reference lists, reviewing the reference lists of relevant review articles, and searching KT+ http://plus.mcmaster.ca/kt/ and EvidenceUpdates http://plus.mcmaster.ca/EvidenceUpdates/, two databases powered by McMaster PLUS [4]. The flow diagram of included and excluded articles is shown in Figure 2.

Reviewer agreement on study eligibility was quantified using the unweighted Cohen κ [5]. The kappa was κ = 0.84 (95% confidence interval [CI], 0.82 to 0.86) for pre-adjudicated pair-wise assessments of in/in and in/uncertain versus out/out, out/uncertain, and uncertain/uncertain. Disagreements were then adjudicated by a third observer.

### Data Extraction

Pairs of reviewers independently extracted the following data from all studies meeting eligibility criteria: study setting, study methods, CCDSS characteristics, patient/provider characteristics, and outcomes. Disagreements were resolved by a third reviewer or by consensus. We attempted to contact primary authors of all included studies via email to confirm data and provide missing data. Primary authors were sent up to two email messages where they were asked to review and amend, if necessary, the data extracted on their study. Primary authors were presented with a URL in the email message. When they clicked on the URL, they were presented with an on-line web-based data extraction form that showed the data extracted on their study. Comments buttons were available for each question and were used by authors to suggest a change or provide clarification for a data extraction item. Upon submitting the form, an email was sent to a research assistant in HIRU summarizing the author's responses. Changes were made to the extraction form noting that the information came from the primary author. We sent email correspondence to the authors of all included trials (n = 168 as of January 13, 2010) and, thus far, 119 (71%) provided additional information or confirmed the accuracy of extracted data. When authors did not respond or could not be contracted, a reviewer trained in data extraction reviewed the extraction form against the full-text of the article as a final check.

All studies were scored for methodological quality on a 10-point scale consisting of five potential sources of bias. The scale used in this update differs from the scale used in the previously published review because only RCTs are included in this update. The scale we used is an extension of the Jadad scale [6] (which assesses randomization, blinding, and accountability of all patients), and includes three additional potential sources of bias (*i.e*., concealment of allocation, unit of allocation, and presence of baseline differences). In brief, we considered concealment of allocation (concealed, score = 2, versus unclear if concealed, 1, versus not concealed, 0), the unit of allocation (a cluster such as a practice, 2, versus physician, 1, versus patient, 0), the presence of baseline differences between the groups that were potentially linked to study outcomes (no baseline differences present or appropriate statistical adjustments made for differences, 2, versus baseline differences present and no statistical adjustments made, 1, versus baseline characteristics not reported, 0), the objectivity of the outcome (objective outcomes or subjective outcomes with blinded assessment, 2, versus subjective outcomes with no blinding but clearly defined assessment criteria, 1, versus, subjective outcomes with no blinding and poorly defined, 0), and the completeness of follow-up for the appropriate unit of analysis (>90%, 2, versus 80 to 90%, 1, versus <80% or not described, 0). The unit of allocation was included because of the possibility of group contamination in trials in which the patients of an individual clinician could be allocated to the intervention and control groups, and the clinician would then receive decision support for some patients but not others. Contamination bias would lead to underestimating the effect of a CCDSS.

### Data Synthesis

CCDSS and study characteristics predicting success will be analyzed and interpreted with the study as the unit of analysis. Data will be summarized using descriptive summary measures, including proportions for categorical variables and means (±SD, standard deviation) for continuous variables. Univariable and multivariable logistic regression models, adjusted for study methodological quality, will be used to investigate associations between the outcomes of interest and study specific covariates. All analyses will be carried out using SPSS, version 18.0. We will interpret p ≤ 0.05 as indicating statistical significance; all p-values will be two-sided. When reporting results from individual studies, we will cite the measures of association and p-values reported in the studies. If appropriate for groups of studies with similar features, we will conduct meta-analyses using standard techniques, as described in the Cochrane Handbook http://www.cochrane.org/resources/handbook/.

## Conclusion

A decision-maker-researcher partnership provides a model for systematic reviews that may foster KT and uptake.

## Competing interests

The authors declare that they have no competing interests.

## Authors' contributions

This paper is based on the protocol submitted for peer review funding. RBH and NLW collaborated on this paper. Members of the Computerized Clinical Decision Support System (CCDSS) Systematic Review Team reviewed the manuscript and provided feedback. All authors read and approved the final manuscript.

## Appendix

Databases searched from 1 January 2004 to 6 January 2010:

### Medline - Ovid

#### Search Strategy

1. (exp artificial intelligence/NOT robotics/) OR decision making, computer-assisted/OR diagnosis, computer-assisted/OR therapy, computer-assisted/OR decision support systems, clinical/OR hospital information systems/OR point-of-care systems/OR computers, handheld/ut OR decision support:.tw. OR reminder systems.sh.

2. (clinical trial.mp. OR clinical trial.pt. OR random:.mp. OR tu.xs. OR search:.tw. OR meta analysis.mp,pt. OR review.pt. OR associated.tw. OR review.tw. OR overview.tw.) NOT (animals.sh. OR letter.pt. OR editorial.pt.)

3. 1 AND 2

4. limit 3 to yr = '2004-current'

Total number of citations downloaded as of January 13, 2010 = 7,578 (6,430 citations retrieved when conducting the search from January 1, 2004 to December 8, 2008; 1,148 citations retrieved when further updating the search to January 6, 2010)

### EMBASE - Ovid

#### Search Strategy

1. computer assisted diagnosis/OR exp computer assisted therapy/OR computer assisted drug therapy/OR artificial intelligence/OR decision support systems, clinical/OR decision making, computer assisted/OR hospital information systems/OR neural networks/OR expert systems/OR computer assisted radiotherapy/OR medical information system/OR decision support:.tw.

2. random:.tw. OR clinical trial:.mp. OR exp health care quality

3. 1 AND 2

4. 3 NOT animal.sh.

5. 4 NOT letter.pt.

6. 5 NOT editorial.pt.

7. limit 6 to yr ='2004-current'

Total number of citations downloaded as of January 13, 2010 = 5,165 (4,406 citations retrieved when conducting the search from January 1, 2004 to December 8, 2008; 759 citations retrieved when further updating the search to January 6, 2010)

### All EBM Reviews - Ovid - Includes Cochrane Database of Systematic Reviews, ACP Journal Club, DARE, CCTR, CMR, HTA, and NHSEED

#### Search Strategy

1. (computer-assisted and drug therapy).mp.

2. (computer-assisted and diagnosis).mp.

3. (expert and system).mp.

4. (computer and diagnosis).mp

5. (computer-assisted and decision).mp.

6. (computer and drug-therapy).mp.

7. (computer and therapy).mp.

8. (information and systems).mp.

9. (computer and decision).mp.

10. decision making, computer-assisted.mp.

11. decision support systems, clinical.mp.

12. CDSS.mp.

13. CCDSS.mp.

14. clinical decision support system:.mp.

15. (comput: assisted adj2 therapy).mp.

16. comput: assisted diagnosis.mp.

17. hospital information system:.mp.

18. point of care system:.mp.

19. (reminder system: and comput:).tw.

20. comput: assisted decision.mp.

21. comput: decision aid.mp.

22. comput: decision making.mp.

23. decision support.mp.

24. (comput: and decision support:).mp.

25. 1 OR 2 OR 3 OR 4 OR 5 OR 6 OR 7 OR 8 OR 9 OR 10 OR 11 OR 12 OR 13 OR 14 OR 15 OR 16 OR 17 OR 18 OR 19 OR 20 OR 21 OR 22 OR 23 OR 24

26. limit 25 to yr = '2004-current'

Total number of citations downloaded as of January 13, 2010 after excluding citations retrieved from Cochrane Database of Systematic Reviews, DARE, CMR, HTA, and NHSEED = 1,964 (1,573 citations retrieved when conducting the search from January 1, 2004 to December 8, 2008; 391 citations retrieved when further updating the search to January 6, 2010)

### INSPEC - Scholars Portal

#### Search Strategy

1. EXPERT

2. SYSTEM?

3. 1 AND 2

4. EVALUAT?

5. 3 AND 4

6. MEDICAL OR CLINICAL OR MEDIC?

7. 5 AND 6

8. PY = 2004:2010

9. 7 AND 8

Total number of citations downloaded as of January 13, 2010 = 87 (84 citations retrieved when conducting the search from January 1, 2004 to December 8, 2008; 3 citations retrieved when further updating the search to January 6, 2010)
